# Improving Conversations about Parkinson's Dementia

**DOI:** 10.1002/mdc3.14054

**Published:** 2024-05-02

**Authors:** Ivelina Dobreva, Joanne Thomas, Anne Marr, Ruairiadh O’Connell, Moïse Roche, Naomi Hannaway, Charlotte Dore, Sian Rose, Ken Liu, Rohan Bhome, Sion Baldwin‐Jones, Janet Roberts, Neil Archibald, Duncan Alston, Khaled Amar, Emma Edwards, Jennifer A. Foley, Victoria J. Haunton, Emily J. Henderson, Ashwani Jha, Fiona Lindop, Cathy Magee, Luke Massey, Eladia Ruiz‐Mendoza, Biju Mohamed, Katherine Patterson, Bhanu Ramaswamy, Anette Schrag, Monty Silverdale, Aida Suárez‐González, Indu Subramanian, Tom Foltynie, Caroline H. Williams‐Gray, Alison J. Yarnall, Camille Carroll, Claire Bale, Cassandra Hugill, Rimona S. Weil

**Affiliations:** ^1^ Dementia Research Centre, Queen Square Institute of Neurology, University College London, Russell Square House London United Kingdom; ^2^ Wellcome Centre for Human Neuroimaging London United Kingdom; ^3^ Central Saint Martins, University of the Arts London United Kingdom; ^4^ UAL Central Saint Martin's London United Kingdom; ^5^ Division of Psychiatry, University College London London United Kingdom; ^6^ Parkinson's UK London United Kingdom; ^7^ Person with Lived Experience; ^8^ South Tees Hospital NHS Foundation Trust Middlesbrough United Kingdom; ^9^ Hillingdon Hospital Uxbridge United Kingdom; ^10^ Royal Bournemouth Hospital, NHS Foundation Trust Bournemouth United Kingdom; ^11^ Livewell Southwest Plymouth United Kingdom; ^12^ Department of Neuropsychology, National Hospital for Neurology and Neurosurgery, Queen Square London United Kingdom; ^13^ UCL Queen Square Institute of Neurology, Univeristy College London London United Kingdom; ^14^ Faculty of Health, University of Plymouth Devon United Kingdom; ^15^ Ageing and Movement Research Group, Bristol Medical School, University of Bristol Bristol United Kingdom; ^16^ Older People's Unit, Royal United Hospitals NHS Foundation Trust Bath United Kingdom; ^17^ Derby Hospitals NHS Foundation Trust Derby United Kingdom; ^18^ National Hospital for Neurology and Neurosurgery London United Kingdom; ^19^ Poole Hospital NHS Foundation Trust Poole United Kingdom; ^20^ North West Anglia NHS Foundation Trust, Peterborough City Hospital Peterborough United Kingdom; ^21^ University Hospital of Wales Cardiff United Kingdom; ^22^ Sheffield Hallam University Sheffield United Kingdom; ^23^ Department of Clinical Neuroscience Institute of Neurology, UCL London United Kingdom; ^24^ Department of Neurology Salford Royal NHS Foundation Trust, Manchester Academic Health Science Centre, University of Manchester Manchester United Kingdom; ^25^ Department of Neurology David Geffen School of Medicine Los Angeles California USA; ^26^ Parkinson's Disease Research, Education, and Clinical Center (PADRECC), Veterans Administration Greater Los Angeles Health Care System Los Angeles California USA; ^27^ Department of Clinical Neurosciences University of Cambridge United Kingdom; ^28^ Cambridge University Hospitals NHS Trust Cambridge United Kingdom; ^29^ Translational and Clinical Research Institute, Newcastle University Newcastle United Kingdom; ^30^ Faculty of Health, University of Plymouth, Drake Circus Plymouth United Kingdom

**Keywords:** Parkinson's, Parkinson's dementia

## Abstract

**Background:**

People with Parkinson's disease (PD) have an increased risk of dementia, yet patients and clinicians frequently avoid talking about it due to associated stigma, and the perception that “nothing can be done about it”. However, open conversations about PD dementia may allow people with the condition to access treatment and support, and may increase participation in research aimed at understanding PD dementia.

**Objectives:**

To co‐produce information resources for patients and healthcare professionals to improve conversations about PD dementia.

**Methods:**

We worked with people with PD, engagement experts, artists, and a PD charity to open up these conversations. 34 participants (16 PD; 6 PD dementia; 1 Parkinsonism, 11 caregivers) attended creative workshops to examine fears about PD dementia and develop information resources. 25 PD experts contributed to the resources.

**Results:**

While most people with PD (70%) and caregivers (81%) shared worries about cognitive changes prior to the workshops, only 38% and 30%, respectively, had raised these concerns with a healthcare professional. 91% of people with PD and 73% of caregivers agreed that PD clinicians should ask about cognitive changes routinely through direct questions and perform cognitive tests at clinic appointments. We used insights from the creative workshops, and input from a network of PD experts to co‐develop two open‐access resources: one for people with PD and their families, and one for healthcare professionals.

**Conclusion:**

Using artistic and creative workshops, co‐learning and striving for diverse voices, we co‐produced relevant resources for a wider audience to improve conversations about PD dementia.

Although Parkinson's disease (PD) is primarily considered a movement disorder, dementia is common, and affects nearly 50% of patients within 10 years of diagnosis.^1^ Mild cognitive impairment, where cognitive deficits are present but do not impact day‐to‐day functioning, is present in 19–42% of newly diagnosed patients.^2^ PD dementia is diagnosed when cognitive impairment becomes severe enough to affect ability to carry out daily activities. It is associated with poorer quality of life and higher caregiver burden compared to PD without dementia,^3^ and double the financial burden compared to other dementias.^4^ Despite the high prevalence and practical implications of PD dementia, there is a lack of awareness of dementia symptoms in people with PD and their families.^5^, ^6^ In a survey of 209 carers, less than half were aware that people with PD are at increased risk of developing dementia.^5^ Many people with PD and their caregivers reported feeling “left in the dark” due to lack of information on dementia, and described current memory services as disjointed, nonspecific and under‐resourced.^7^ Of concern, in a survey of 74 UK PD experts and healthcare professionals, only 14% said their training had prepared them to provide high‐quality care for people with PD‐related dementia.^5^ Cognitive function in PD was selected as a “community priority of major unmet need” by the PD Foundation Community Choice Research Award Program,^8^ and lack of discussion about cognitive change was emphasized in a working group of experts convened in response.^8^ Barriers to conversations about dementia in the wider population are: (1) the stigma of a dementia diagnosis, including fear around the diagnosis, and worry about social exclusion; (2) the perception that treatment options are limited; and (3) that there is little benefit in early diagnosis of dementia.^9^ These factors are likely to be even greater in PD, where there is already a perceived stigma of the diagnosis of PD itself.^10^

Barriers to help‐seeking for cognitive problems are multifaceted and may be more common in Black and other minoritized ethnic populations.^11^ In a recent study, Black African and Caribbean community members described dementia as “a white person's illness”, expressed views about futility of seeking help for cognitive symptoms, and raised concerns about maintaining personal affairs private.^12^ A review of experiences of dementia in people of Black African and Caribbean backgrounds highlighted dissatisfaction due to inappropriate and perceived disrespectful treatment as a barrier to help‐seeking.^13^ Studies in South Asian communities also reported barriers to accessing services, including stigma associated with dementia, lack of culturally appropriate services, and preference for culturally‐aligned coping strategies.^14^ To avoid stigma for the family, members from South Asian communities may provide care for affected family members privately, rather than seek medical or social care assistance.^14^

There are also challenges to discussing dementia in the clinic for PD healthcare professionals. A recent multidisciplinary symposium on unmet needs in cognitive health in PD found that cognition is not routinely or consistently assessed in the PD clinic, in contrast to motor function.^8^ Barriers to clinicians initiating conversations about PD dementia include a perception that there is nothing that can be done about it; lack of confidence in having these conversations^5^, ^15^; lack of standardized ways to assess cognition in PD^8^; fear of inducing worry in people with PD or PD dementia^15^; and lack of time during appointments.

However, if dementia is not discussed in the PD clinic, people with PD dementia will not receive appropriate treatment or access support that is vital for them and their families. Receiving an earlier diagnosis of dementia allows people with PD and their families to prepare for the future. Creating opportunities to discuss dementia‐related issues can also engage patients in research and clinical trials in the PD dementia field.

To address the challenge of talking about dementia in PD, we worked together with people with PD and their families to (1) identify roots and triggers of discomfort linked with talking about dementia; and (2) to find out how and when people wanted to hear about dementia in the clinic. We examined these issues during creative workshops, using visual‐art methods, with the aim that these would help support challenging conversations.

There is an increased recognition of the benefits of creative visual expression generally, with research documenting the benefits of art for improved emotional awareness in acute psychosis,^16^ and improving psychological stability and reducing anxiety in people with coronary heart disease.^17^ Creative thinking has specifically been linked with PD, especially with dopaminergic therapy.^18^ Creative approaches have previously been applied as research methods to facilitate challenging conversations. For example, an artist‐educator was employed to co‐produce artwork to engage ethnic minorities in group discussions about clinicals trials^19^; and visual art‐based approaches were used to communicate sexual and reproductive health behavior in young South African women.^20^ Both these projects demonstrate the benefits of creative and visual‐art approaches to facilitate challenging conversations, with the visual cues from the art providing prompts for discussion. We therefore worked together with artists to facilitate challenging conversations around dementia in PD. This project utilizes novel creative visual arts methods to facilitate conversations on dementia in PD.

We took several steps to ensure we included a diverse range of voices of lived experience, so our findings would be more widely applicable. We used outputs from these workshops to form the basis of a pair of information resources to support dialogue and awareness of PD dementia: one for people with PD and their families; and one for healthcare professionals to support these conversations in the clinic. We then held a series of focus groups and received feedback from people with lived experience and 25 PD experts to co‐develop and refine these resources further. Both resources are now openly accessible as part of Parkinson's UK's information resources^21^, ^22^ (See supplemental material).

We describe here the process of co‐developing these information resources to ensure they would be widely accessible, well‐targeted and practical.

## Methods

The project involved three stages

1. Creative workshops with people with PD, PD dementia and their caregivers, to identify roots and triggers of discomfort around conversations about PD dementia; and to find out how, and when, people would like to receive information about PD dementia in the clinic. The outputs of these workshops formed the first draft of the information resources.
2. Focus groups with people with PD, PD dementia and their families, and with PD experts, to refine the information resources.
3. Additional consultation to access further feedback and input into the resources from PD experts and the Parkinson's community.

### Participants

We recruited participants between May and June 2022 from PD clinics at the National Hospital for Neurology and Neurosurgery, including clinics led by a member of the study team (RSW) and from a database of patients from PD clinics at the same hospital who had previously expressed an interest to take part in research. We also recruited participants who had previously taken part in research studies at our center, and from Parkinson's UK, and community information events about research participation. We made efforts to ensure we included a diverse range of people with PD. To achieve this, we included a researcher on our core team (MR) who works on the experience of dementia in Black African, Caribbean, and other minoritized ethnic communities. MR guided our recruitment plans and materials and provided input throughout every stage of the project. Our recruitment goal was at least 50% Black and other minoritized ethnic participants. Inclusion criteria were diagnosis of PD (or parkinsonism) and their carers/partners. We aimed to involve people with PD at different stages of progression: some with no cognitive involvement, and others who already had cognitive symptoms or a PD dementia diagnosis. Dementia and cognitive capacity for inclusion was assessed at the screening stage, and during the consenting process, using standard processes established in the UK Mental Capacity Act. There were no exclusion criteria, but there were conditions which may have prevented participation (eg, other medical or psychiatric conditions interfering with participation). We approached 55 people with PD at different stages of progression, and their carers (response rate 61%). Reasons for non‐participation included deteriorating health precluding travel to the venue (*n* = 7), availability for workshop dates (*n* = 6), and no response to contact (*n* = 8). Of those who agreed to participate, 15 had a pre‐existing relationship with the team involved in the workshop (clinic patients or participants from previous research studies).We collected basic demographic information, including ethnicity and diagnosis..

### Stage 1: Creative Workshops

We divided participants into two groups: predominantly PD without dementia (total *n* = 14; comprising *n* = 9 people with PD; and *n* = 5 caregivers); and predominantly people with PD dementia (total *n* = 20; comprising *n* = 13 people with PD and *n* = 7 caregivers). These groups aimed to allow participants to discuss attitudes towards dementia with others at a similar disease stage. We were limited by participant availability on workshop days, so each group had some people with PD and PD dementia. Each group attended two workshops over two months, which were held at Central Saint Martin's School of Art (part of University of the Arts London). Four participants dropped‐out between workshops due to deteriorating health (*n* = 2) and excessive summer heat (*n* = 2). Workshops were designed by the core team which included artists experienced in using creative methods to facilitate conversation; a person with lived experience of PD; PD clinicians and researchers; and public engagement experts. Each workshop lasted approximately 120 min, and included a brief ice breaker session with a gentle physical warm‐up led by a Pilates instructor and a short drawing exercise, intending to help participants begin collaboration and conversation. This was followed by the core creative session (45 min) and a “show and tell” discussion facilitated by two artists in the core team (45 min).

During all workshops, clinicians, researchers, and public engagement experts participated in the creative elements. On‐site breakout‐rooms and nominated staff members were available if participants felt distressed during discussions, but were not required. Support information from Parkinson's UK in the form of the charity website and hotline, was also shared during and after workshops.

Workshop 1 explored beliefs, fears and attitudes people with PD and their family members held about dementia. Using paint, drawing and collage, participants shared their associations with PD dementia. Through these media, they were encouraged to explore their attitudes towards receiving a diagnosis and consider perceptions about living with PD dementia. This included any difficulties in talking to others about the diagnosis. Participants were invited to create as many artistic outputs as they wished. After the creative session, participants provided descriptions of their artwork in a “show and tell” discussion.

In Workshop 2, participants were invited to create artworks using paint and collage to share how they would like to learn about dementia from healthcare professionals. Key questions were: “How would you prefer to learn about dementia?”; “At what stage?”; “From whom?”; “Where?”; “In what format?”; and “What does excellent PD dementia support look like?”. As in the first workshop, participants were encouraged to create as many outputs as they wished and provided descriptions of their artwork.

**Figure 1:**
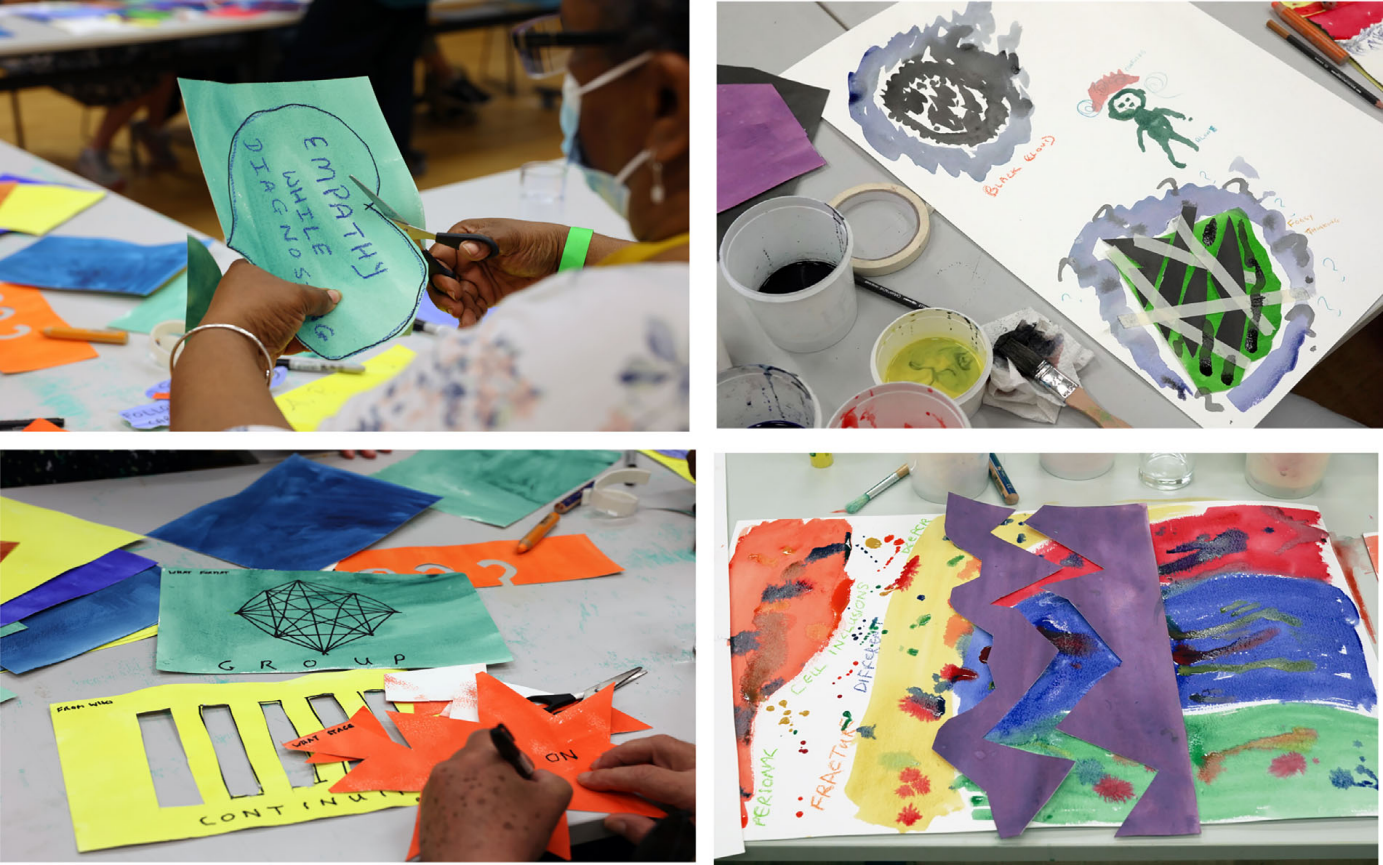
Outputs from the creative workshops that informed the content of information resources.

During the “show and tell” session of each workshop, discussions and comments were transcribed by two members of the team. See Figure 1 for creative outputs from the workshops.

Themes from the workshops were used to guide development of a pair of information resources: one for people with PD and their families and one for healthcare professionals.

### Thematic Analysis of Themes from Workshops

An inductive thematic analysis of notes and artwork was conducted.^23^ After initial familiarization with the data, NVivo software (version 14.23.2) was used to independently code the available data from notes taken during the artistic workshops. We used an iterative process, with multiple readings and successive coding and recoding of the data. This was performed individually by two researchers (ID and NH), and then discussed together until a consensus on coding was reached. Codes were then grouped according to similar categories to create themes. The emerging themes were further reviewed, modified and re‐defined until final themes emerged.

### Survey on Dementia Attitudes and Participation Feedback

Participants completed an anonymized questionnaire of open and closed‐ended questions exploring attitudes and fears regarding PD dementia before each workshop. We additionally asked participants to complete an anonymized questionnaire with open and closed‐ended questions about change in attitudes to PD dementia following participation; and about their experiences during the project (see Supplement for questionnaires). An informal analysis of themes arising from attitudes to dementia was taken into account when designing the resources. Feedback on workshop participation did not directly inform the information resources.

### Stage 2: Focus Groups

We held a series of focus groups with workshop participants, to refine and review the information resources, which had been developed using creative outputs and discussions during the workshops. Two groups of participants, remaining in the same groups as the workshops, each took part in three focus groups. The groups were: PD without dementia (total *n* = 6; comprising *n* = 6 people with PD); and people with PD dementia and their caregivers (total *n* = 8; comprising *n* = 2 carers and *n* = 6 people with PD dementia). The focus groups were led by a moderator (JT) and observer (ID). We used open questions to avoid biases in the responses. In the first session, we started by seeking feedback on the resource in general, (eg, “What do you think about the information resources?”; “What works? And what doesn’t work?”). This was followed by more focused questions to resolve specific issues on the content. In the second and third sessions, we sought feedback on overall appearance, design, and structure of the information resources as well as a review of specific sections of the content. This enabled us to receive lived experience feedback on how to communicate key issues such as quantification of risk; prevention strategies including exercise; and advanced care planning.

In addition, we held two focus groups, led by a moderator (RW) and two observers (JT, ID) with a total of 12 healthcare professionals (comprising two PD nurses, one psychologist, three geriatricians, six neurologists), recruited from PD professional networks. Their purpose was to review and co‐develop each of the information resources, and ensure information reflected current best practice. Specifically, for the patient‐focused information resource, open‐ended questions were employed to gain feedback on the communication of prevalence and risk factors for PD dementia, inclusion of advice about exercise, feedback on design, and how the resource might be used in the clinical setting. For the healthcare professional resource, we asked questions that sought feedback on the guidance on cognitive assessment, diagnostic procedures, treatment strategies, and overall tone of the resource. For each focus group, 1–2 people from the team transcribed the discussion, so that this could be reflected upon afterwards.

After each focus group, the moderator and observers reflected on the feedback and incorporated suggested edits, where possible. Both resources were refined in an iterative process, with edits being introduced after each focus group, and updated versions of the resources presented for further review in subsequent focus groups. A final draft of both resources was distributed to all healthcare professionals, and all workshop participants, after feedback had been implemented.

### Stage 3: Additional Consultation

A final stage was to test the near‐final draft booklets with people who had not yet been involved in the project, to ensure the content was widely accessible, complete, and relevant. We held a focus group with people with PD (*n* = 8), recruited through Parkinson's UK, who had not attended the workshops. Participants were provided with the patient‐facing resource prior to the session. At the focus group, led by a moderator (JT) and an observer (ID), they were asked for feedback on resource content, utility, use of language, format, and layout.

We sought opinions from an additional 13 PD healthcare professionals to review the information resources, check factual content and advice, and consider the way information was communicated. We included eight neurologists, one psychologist, two physiotherapists, an occupational therapist, and one psychiatrist in this feedback stage.

To measure impact of the resources, we collected data on number of online downloads, and number of requests for printed versions in the three months following release.

## Results

34 people affected by PD took part in the four creative workshops. These included 16 people with PD, diagnosed according to UK Brain Bank criteria, six with PD dementia (diagnosed clinically,^24^ one person with parkinsonism, 11 family members or caregivers (nine spouses and two siblings). Mean disease duration was 6.76 years, SD = 3.96. None of the people with PD had a formal diagnosis of PD‐MCI. We included a person with parkinsonism who was going through a diagnostic process, as this reflects the experience of people with PD, who may receive a definitive diagnosis at a later stage. 43% of people with PD and 46% of caregivers self‐identified as Asian, Black, or Mixed ethnic group. In addition, two clinicians, four researchers, four public engagement experts, and four art facilitators participated in the workshops. Characteristics of participants affected by PD are presented in Table 1.

**TABLE 1:** Demographic data

Attribute	People with Parkinson's *n* = 23	Caregivers *n* = 11
Age, years	69.1 (5.6)	69.5 (6.2)
Male, *n* (%)	14 (60.68%)	0
Female, *n* (%)	9 (39.13%)	11 (100%)
Ethnicity, *n* (%)		
Asian	7 (33.3%)	1 (9%)
Black	2 (9.52%)	3 (27.27%)
Mixed	0	1 (9%)
White	12 (57.14%)	6 (54.54%)
Not disclosed	2 (9.52%)	0
Time since Parkinson's diagnosis, years	6.76 (3.96)	N/A

Age and time since diagnosis variables shown in mean (SD). Other data reported in number of participants, and percentage.

### Themes Emerging from Workshops

Three main themes were identified from creative outputs and discussions in workshop 1:

(1) fear of PD dementia symptoms and their unpredictability; (2) stigma associated with receiving a diagnosis, including a worry of exclusion from communities; and (3) the importance of support in adapting to the changes dementia diagnosis brings.

Words linked with dementia included “scared” and “struggle”. Participants expressed fear over loss, specifically loss of identity, control, independence, and social life. People also described fear of not being seen as an individual, but as a “person with a disease”, and the feelings of loneliness this might bring.

Stigma emerged as tightly linked to fear, with participants describing their reluctance to share receiving the diagnosis with friends or extended family. In particular, they expressed a worry about being categorized or “put in a dementia basket” and how they would be perceived in their community.

Finally, participants commented on the importance of receiving timely care and support from medical professionals, in adapting to changes a dementia diagnosis brings. The support network from communities, and connections with others was highlighted, especially in the context of overcoming feelings of loneliness.

Workshop 2 explored how participants would like to receive information about PD dementia. The majority of participants described that they would like to receive this information from a specialist medical professional during a longer consultation time, early in the disease journey, and have the opportunity to take home a written resource that is easily accessible and that they can refer to. They also expressed the need for clear information on medication and how it can help in the context of PD dementia, and guidance on available support.

There were occasional dissenting responses which did not fit the common themes. Two participants had strong religious and spiritual opinions, and one person preferred conversations about dementia not to be held in clinic before symptoms appear, to avoid unnecessary worry.

### Survey Results on Attitudes to Dementia

Prior to the workshops, 70% of people with PD and 81% of caregivers shared that they had been worried about PD dementia. However, only 38% of people with PD and 30% of caregivers had previously raised these concerns with a healthcare professional. Reasons for not previously raising these worries were lack of opportunity, and fear of discussing the issue in front of their loved one (See Box 1).

Box 1 **Descriptions of barriers to talking about PD dementia**

“I did not like to raise it [with a doctor] in front of my partner” (Caregiver)

“I volunteer with a wider PD group and while everything else is discussed, dementia isn't. We are a very social group, and it would be very helpful to have a new way of talking about dementia without scaring everyone.” (Caregiver)

“I felt my consultant did not want to address the issue of dementia” (Participant with PD dementia)

[We had a] “good discussion, but short – only 10 minutes” (Participant with PD dementia)

“Fear – I am a Christian and I believe that what you talk about is what you get. I believe in God protecting my husband [from dementia]” (Caregiver)

Experiences of discussing dementia with healthcare professionals varied from “constructive”, to “not very helpful”. Caregivers found the conversation “quite easy but concerning”, “distressing, although expected diagnosis of dementia”, and “very helpful”.

When asked if there was anything about how the PD dementia diagnosis could have been improved, one person with PDD and one caregiver wanted more information, one person with PDD did not wish to receive a diagnosis; and one felt their consultant was reluctant to discuss it. Three people with PDD responded that nothing could be improved.

90% of people with PD and 73% of caregivers agreed that clinicians should ask people with PD about cognitive changes routinely. They indicated this should be done through direct questions, routine cognitive tests, and more regular appointments to track changes (see Box 2).

Box 2 **How people with PD want to talk about PD dementia.**

“I prefer a direct question with a preamble by the clinician of how it is routine”. (Participant with PD)

“Point out what it can be like [when cognitive symptoms occur] to enable us to identify the issues early on”. (Participant with PD)

“Important to allow sufficient time to fully discuss the issue”. (Participant with PD)

“Ask routinely and by explaining what symptoms may occur to bring awareness”. (Participant with PD)

“Reviews need to be more regular, as we currently see our Parkinson's nurse [only] once per year”. (Participant with PD)

“Sensitively, perhaps together with caregivers, as they would notice some early changes”. (Participant with PD)

“Provide more explanations about possible symptoms and what further treatments or help there is”. (Caregiver)

Two people highlighted the need for more time during appointments, and importance of caregivers in recognizing early changes. When asked about different words to substitute for “dementia”, most people did not mind the word “dementia” in facilitating conversations around cognition.

### Survey Results on Experience of Participating in the Creative Workshops

Feedback from people with PD and caregivers on participating in the creative workshops was overwhelmingly positive. They used the words “emotional”, “enlightening”, “heart‐warming”, “supportive”, and “brilliant” to describe how it felt to take part (see Box 3). They described it as reassuring to hear other people's similar experiences and learn from them. 80% of people shared that participating in the creative workshops had changed the way they view conversations on PD dementia. Participants described that they are now more “open to” and “confident in” holding these conversations and would find it easier to talk about this with their family or clinicians. All participants (100%) responded that they would take part in a similar project again, or would recommend taking part in a similar project to someone they know.

Box 3 **Feedback on taking part in creative workshops to improve PD dementia conversations.**

“Prior to [the] workshop I couldn’t quite understand how art could be used to discuss dementia – I was quite inspired and enlightened”.

“It has been eye opening to see that people are all so different [in the way they experience PD] and see things so differently and yet so coincidentally”.

“Being part of this project helped me understand that I am not alone in living with PD—others are going through the same experiences, and we can learn from each other”.

“Joining in the workshops brought a feeling that I was contributing to something that would be of real, tangible benefit to many”.

“I have learnt not to be afraid of talking about my feelings of dementia. Meeting others with similar experience definitely helps in making it easier to convey”.

### Content Co‐Development for the Information Resources

We used outputs from the workshops, and the surveys as the basis of the two information resources: one for people with PD and their families, and one for healthcare professionals.

We structured the content of the patient information resource into nine sections, beginning with what cognitive changes are like in PD, and why they happen. We included a section on how to raise worries about dementia with a specialist; information on medications; and details of support for caregivers, including advanced stages and end of life.

**Figure 2:**
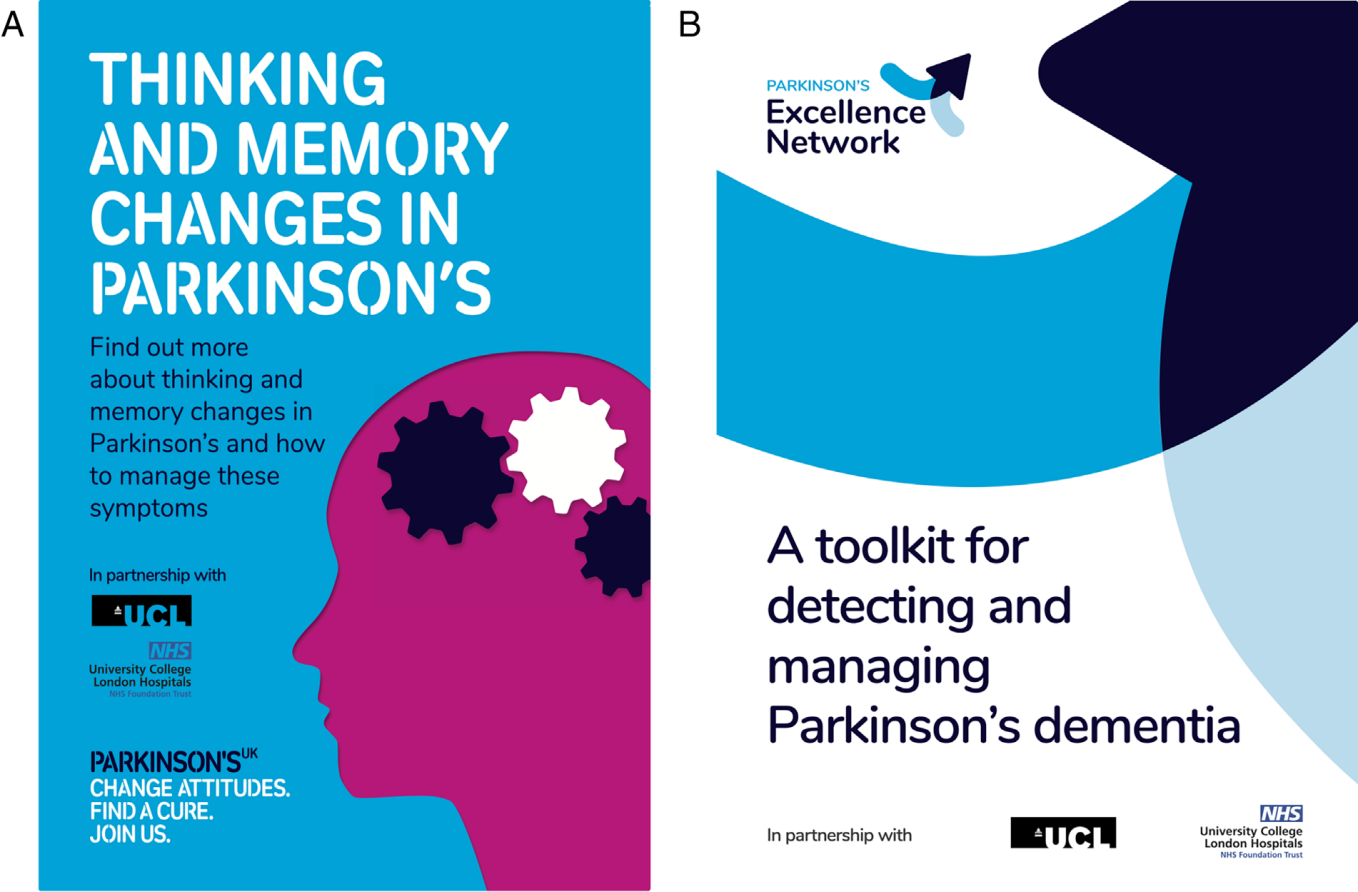
Front covers of the information resources for (**A**) people with PD; and (**B**) for healthcare professionals.

The healthcare professional resource was designed to be of practical use in a clinic where time may be limited, with information on recognizing and diagnosing PD dementia, and practical management guidance (see Fig. 2 for cover images of information resources).

Both information resources have been adopted as resources on PD dementia by Parkinson's UK, and are freely available to download.^14^, ^15^ Within 3 months of their launch, pages with these resources have already had 17,869 page views (13th most viewed page on Parkinson's UK's site in that time period). Print orders for the patient booklet is 769, and 334 for the healthcare professional resource.

## Discussion

In this collaborative project, we aimed to identify roots and triggers of discomfort linked with dementia within the PD community and to co‐produce a pair of information resources, for people with PD and healthcare professionals, to improve conversations about PD dementia. We found that although 70% of patients and 81% of caregivers had been worried about PD dementia, only 38% and 30%, respectively, had raised these concerns with a healthcare professional. Conversely, we found that during the workshops, people affected by PD were willing to talk and learn about dementia, and were open to discussing risk of dementia and available support. Even though people with PD acknowledged that talking about dementia can be challenging, they recognized the benefits of doing so and most thought PD clinicians should routinely and directly ask patients about cognitive changes and use cognitive tests at regular clinic appointments.

Discussing dementia in a PD clinic where time is often limited, can be challenging. Conversations may be hindered by barriers for people with PD and healthcare professionals. We found using creative approaches helped people speak more freely and broach conversations they might have found difficult to bring up in other settings. Whilst this would not usually be practical in clinical settings, it allowed us to explore these issues and develop useful information resources. These will mean that people with PD dementia can now access information on risk, timely diagnosis, treatment, and patient‐centered support.

Working with artists and using a creative process for the workshop stage, enabled people with PD to feel comfortable and encouraged participants to open‐up in a way they may have found challenging in more conventional settings. Delivering workshops away from the hospital, together with clinicians and researchers, helped to reduce usual patient‐doctor hierarchies, and ensured clinicians and researchers became more equal partners in this project.

There is increased recognition of the utility of arts‐based interventions (including art‐making and dance) for people with dementia and their caregivers. Art‐making was linked to increased well‐being, and improved social participation and psychological health.^25^, ^26^ Creative expression and artistic activities such as painting are also an important way for people with dementia to express emotions even in the presence of cognitive decline.^27^, ^28^ In PD, art‐based interventions showed improvements in motivation and creativity,^29^ and participation in dance programs leads to improvement in motor symptoms and well‐being.^30^, ^31^ This project therefore used a novel creative arts process to facilitate conversations about PD dementia.

Using workshop and focus group outputs, we created two information resources, which benefited from the input of PD experts from a range of clinical settings around the UK. This meant we had the most up‐to‐date guidance on diagnosing and managing PD dementia, from experts across a range of disciplines. By disseminating these tools through Parkinson's UK (the largest UK charity for PD), we can ensure they will be freely available, widely accessible and viewed as trustworthy and credible sources of information. It will also provide a framework for updates as new guidance and information becomes available for PD dementia.

### Strengths and Limitations

Particular strengths of this project are that the voices of people with PD were at the core of all stages; and that we included a diverse range of people with PD, particularly those from ethnic minority backgrounds who are often underrepresented in PD research. This meant our resources could resonate with more people with PD, be useful to a wider audience, and that we could address issues, such as the stigma of PD dementia, that might not be raised with a more homogeneous group.

Although literature exists on PD dementia for people living with PD and their families, our resources provide three distinct and novel benefits: firstly, they were co‐produced with people with lived experience who provided their perspective. Secondly, the resources include key practical information around support and planning. Finally, they have been produced as a complementary pair: with one resource for people with lived experience, and one for healthcare professionals.

We aimed for diversity on a range of aspects, however, this project was carried out in the UK. Factors such as cultural views on dementia, access to healthcare, and support infrastructure will vary in other parts of the world. Our group was relatively young for PDD, with relatively short disease duration, so may not be fully representative of populations with PDD. It was also likely to be a self‐selecting group who might be more comfortable talking about dementia than the general PD population. Our workshops were intentionally small, to allow people to feel comfortable talking about dementia in a friendly environment. However, this meant that our sample size was relatively small, in common with qualitative research in general. Surveys or questionnaires sent anonymously can reach larger populations, and potentially provide input from diverse geographical areas, but have less ability to engage participants in the questions at hand and enable the same depth of discussion as smaller group engagement. Ultimately both types of engagement: creative and local, as well as larger surveys, can provide complementary input into these important questions. Future work could apply similar approaches with a more global outreach, to develop more international guidance.

Some of the workshop participants (*n* = 15) had a pre‐existing relationship with the study team (clinic patients, or participants in previous research studies) which could have influenced their responses during open discussion. We sought to minimize this effect by anonymizing survey questionnaires. We had planned to hold separate workshops and focus groups for people at different stages of the dementia journey, but this was not possible due to scheduling constraints of participants. In practice, we found that having groups that were mixed in their experience of dementia was not a barrier to active discussion, and the contributions to the information resources were more joined‐up as a results of some mixing of the groups.

Given the creative nature of the workshops, some motor problems may have presented as limiting factors during the art‐making exercise. In practice, the artistic outputs included simple painting with comfortable brushes, drawing and collage making, and assistants were available where required. Participants were all able to express their viewpoints. Educational and sociodemographic background of the participants, which could have an influence on conversations about dementia, was not collected, and future work should include this. We note that all carers were female. PD is more common in men, with an even higher male predominance for PDD^32^ and this is reflected in the higher prevalence of males living with PD and PDD in our sample. However, it is notable that females living with PD or PDD either attended alone or with a female carer. Carer participation has previously been noted to be higher in other diseases, for example in cancer^33^; and in a meta‐analysis, caregivers are more likely to be female, and women provide more hours of caregiving, and experience higher levels of care giver stress than men.^34^ Future work should aim to include carers of both genders if possible.

## Summary

Through a process of artistic workshops, co‐learning and bringing in diverse voices, we co‐developed two information resources to improve conversations about PD dementia: one for people living with PD, and one for healthcare professionals, that are freely available to be used by people with PD and healthcare professionals. We hope that these resources will help facilitate these challenging conversations and result in helping people living with PD dementia to access more timely diagnosis, treatment, and patient‐centered support.

## Authors Roles

(1) Project: A. Conception, B. Organization/Recruitment, C. Execution; (2) Statistical analysis: A. Design, B. Execution, C. Review and Critique; (3) Manuscript: A. Writing of the first draft, B. Review and Critique.

I.D.: 1B, 1C, 2A, 2B, 3A

J.T.: 1A, 1B, 1C, 3B

A.M.: 1A, 1B, 1C, 3B

R.O.: 1A, 1B, 1C, 3B

M.R.: 1C, 1B, 3B

N.H.: 1C, 3B

C.D.: 1C, 3B

S.R.: 1C, 3B

K.L.: 1C, 3B

R.B.: 1C, 3B

S.B.J.: 1C, 3B

J.R.: 1A, 1C, 3B

N.A.: 1C, 3B

D.A.: 1C, 3B

K.A.: 1C, 3B

E.E.: 1C, 3B

J.F.: 1C, 3B

V.H.: 1C, 3B

E.H.: 1C, 3B

A.J.: 1C, 3B

F.L.: 1C, 3B

C.M.: 1C, 3B

L.M.: 1C, 3B

E.R.M.: 1C, 3B

B.M.: 1C, 3B

K.P.: 1C, 3B

B.R.: 1C, 3B

A.S.: 1C, 3B

M.S.: 1C, 3B

A.S.: 1C, 3B

I.S.: 1C, 3B

T.F.: 1C, 3B

C.H.W.G.: 1C, 3B

A.Y.: 1C, 3B

C.C.: 1C, 3B

C.B.: 1A, 1C, 3B

C.H.: 1A, 1C, 3B

R.S.W.: 1A, 1B, 1C, 2C, 3B

## Disclosures

**Ethical Compliance Statement:** The study was approved by the London Chelsea NHS Research Ethics Committee. Participants provided written informed consent before participating. See supplemental data for GRIPP checklist considering patient‐public involvement for this study. We confirm that we have read the Journal's position on issues involved in ethical publication and affirm that this work is consistent with those guidelines.

**Funding Sources and Conflicts of Interest:** This project was funded by a Wellcome Research Enrichment—Public Engagement Grant (205,167/Z/Z16/A).

No direct financial support was received from Parkinson's UK. However, the project received in‐kind support through graphic design of the information resources. RSW is supported by a Wellcome Clinical Research Career Development Fellowship (205167/Z/16/Z). NH is supported by a grant by the Rosetrees and Stoneygate Trusts. CHWG is supported by a Transition Fellowship from the Medical Research Council (MR/W029235/1) and by the NIHR Cambridge Biomedical Research Centre (NIHR203312; the views expressed are those of the authors and not necessarily those of the NHS, the NIHR or the Department of Health). AM has received workshop consultancy honoraria from Crossover Labs. EE reports Honorariums from Bial and Neurology academy. JAF has received research funding from Parkinson's UK. VJH has received travel support from Bial and research funding from Parkinson's UK and National Institute for Health and Care Research. EJH has received honoraria and/or travel support/advisory board contribution from Kyowa Kirin; Simbec Orion, Abbvie; Ever; Bial; and the Neurology Academy; and research funding from The Gatsby Foundation, Royal Osteoporosis Society, National Institute of Health Research and Parkinson's UK. CWG has received grant support from Cure Parkinson's, Parkinson's UK, the Rosetrees Trust, and consultancy fees from Evidera. AJY has received honoraria from GE Healthcare, plus grants from Cure Parkinson's, Lewy Body Society, EU IMI, Parkinson's UK, Dunhill Medical Trust, NIHR. RSW has received speaking honoraria from GE Healthcare and Bial, and has provided consultancy to Therakind. The authors declare that there are no conflicts of interest relevant to this work.

**Financial Disclosures for the Previous 12 Months:** The authors declare that there are no additional disclosures to report.

## Supporting information


**Supplementary Material** **:** Section A. Checklist on reporting public involvement in health research (GRIPP2 short form).
Section B. Open access links to Parkinson's dementia resources.
Section C. Workshop and feedback surveys.

## Data Availability

The data supporting the findings of this study are available from the corresponding author, upon reasonable request.
